# Number of pain points and multisite pain in people 80 years and older in Thailand

**DOI:** 10.1097/PR9.0000000000001375

**Published:** 2025-12-12

**Authors:** Supa Pengpid, Karl Peltzer, André Hajek, Razak M. Gyasi

**Affiliations:** a Department of Health Education and Behavioral Sciences, Faculty of Public Health, Mahidol University, Bangkok, Thailand; b Department of Public Health, Sefako Makgatho Health Sciences University, Pretoria, South Africa; c Department of Healthcare Administration, College of Medical and Health Science, Asia University, Taichung, Taiwan; d Department of Psychology, University of the Free State, Bloemfontein, South Africa; e Department of Psychology, College of Medical and Health Science, Asia University, Taichung, Taiwan; f Department of Medical Research, China Medical University Hospital, China Medical University, Taichung, Taiwan; g Department of Health Economics and Health Services Research, University Medical Center Hamburg-Eppendorf, Hamburg Center for Health Economics, Hamburg, Germany; h African Population and Health Research Center, Nairobi, Kenya; i National Centre for Naturopathic Medicine, Faculty of Health, Southern Cross University, Lismore, NSW, Australia

**Keywords:** Adults 80 years and older, Number of pain sites, Multisite pain, Longitudinal study, Thailand

## Abstract

**Introduction/Objectives:**

The factors influencing the number of pain sites (NPS) and multisite pain (MSP) in adults 80 years and older have not been thoroughly examined in many research. The purpose of this study was to estimate these drivers using Thailand's 3-wave national longitudinal data.

**Methods:**

We used data of people 80 years and older (analytic sample: n = 2450 observations) from the 2015, 2017, and 2022 Health, Aging, and Retirement in Thailand survey. Number of pain sites and multisite pain were measured using established metrics. The time-variant causes and outcomes were evaluated using fixed-effects regression.

**Results:**

The average NPS was 2.4 (SD = 3.1); the proportion of MSP was 46.1% and 31.4% for having ≥2 and ≥3 pain sites, respectively; and 42.8% reported moderate or severe pain on at least 1 pain site. Fixed-effects (FE) regressions showed that increasing age, subjective economic status, and work status decreased the NPS. Transitioning to live alone, past smoking, and an increase in depressive symptoms increased the NPS. Regarding FE regressions with MSP (≥2 and ≥3 pain sites), age and subjective economic status decreased the odds of MSP, transitioning to live alone, past smoking, depressive symptoms, and the number of chronic conditions increased the odds of MSP.

**Conclusions:**

Decreasing age, decreasing subjective economic status, not working, living alone, past smoking, depressive symptoms, and chronic conditions were associated with the NPS and/or MSP.

## Abstract 2

The average number of pain sites was 2.4 (SD = 3.1), multisite (≥3) pain was 46.1%, and 42.8% reported moderate/severe pain on at least 1 pain site.

## 1. Introduction

Thailand's population is rapidly aging, similar to that of many low- and middle-income nations.^2^ In Thailand, 1.4 million persons, or 1.9% of the total population, are 80 years or older. According to the World Data Atlas,^34^ this number is predicted to increase rapidly at a rate of 7% each year, reaching roughly 3 million by 2039.^11^ Significant declines in the main causes of death have been linked to Thailand's longer life expectancy.^28^ Compared with younger adults, older adults experience more chronic pain and are more likely to experience pain at many sites due to the higher prevalence of pain-causing illnesses (such as diabetes and arthritis).^31^ Yet, there is a limited understanding of the prevalence and correlates of pain in the oldest-old adults,^6^ particularly in low- and middle-income settings.^13^ This knowledge would be relevant because pain in older adults and/or the oldest-old has been strongly linked to excess disability,^14^ insomnia, depression,^9^ increased healthcare utilization,^30^ and future fall risk.^33^

The prevalence of pain in oldest-old adults includes, eg, mild or severe pain of 68%, multisite pain (MSP) 46%, and severe pain in at least 1 body location 35% for individuals 85 years and older in Sweden.^7^ Again, in North Rhine-Westphalia, Germany, 45.1% of the oldest-old adults had moderate pain and 26.4% severe pain,^6^ and in a multisite study in Germany, 33% experienced mild to severe pain and 69% mild to significant impairment owing to pain with everyday activities.^17^

Factors associated with pain in the oldest-old adults include age. For example, in Sweden, total reported pain decreased with age among women, and severe pain increased with age among men,^7^ while in Germany, compared with 80 to 84-year olds, being 85 years or older decreased moderate pain, and being 85 to 89 years old decreased severe pain^6^; and the multisite study in Germany showed a decline in pain at very old age.^17^ Other sociodemographic factors associated with pain in oldest-old adults include female sex,^6,17^ lower education,^6^ and living at home with care support.^17^

Health-related factors associated with pain in oldest-old adults include poorer self-rated health status, a higher number of chronic diseases,^6^ multimorbidity,^18^ frailty,^24^ use of analgesics, and depression.^17^ In a systematic review of older adults involving 8 studies in the meta-analysis, overweight and obesity are both associated with chronic pain.^29^ In an 11-year follow-up study among adults, former and current smoking status were associated with an increased risk of chronic widespread pain (CWP)^22^ and number of pain sites (NPS),^21^ and high and moderate levels of alcohol intake were related with a lower risk of CWP.^22^

Based on national, longitudinal community-dwelling data in Thailand from 2015 to 2022, our study aimed to explore the determinants of NPS and MSP among the very old (80 years and older) (also stratified by sex) in light of the paucity of data on this topic, particularly in relation to longitudinal studies in low- and middle-income countries and exclusively among the oldest-old. Developing therapies to help the very elderly requires an understanding of the factors that lead to NPS and MSP.

## 2. Methods

### 2.1. Participants and procedures

Analysis was performed on 3 waves of health, aging, and retirement in Thailand (HART) investigations that were conducted in 2015, 2017, and 2022. One adult (older than 45 years) was randomly selected in each household (as the inclusion criterion) and questioned in the home using a multistage national sample method; additional information is provided elsewhere.^3^ Only participants who were 80 years or older were included in the sample for this research; 2450 observations from 3 research assessments in 2015, 2017, and 2022 made up the analytic pooled sample. Of the 1038 participants at baseline, 335 were untraceable, 5 declined, and 37 had passed away. The response rate was 66%. Key sociodemographics (age, education, economic status, living alone, residence, employment, and marital status) and health-related variables (number of chronic conditions, NPS, MSP, and self-rated physical health status) did not significantly differ among participants lost to follow-up compared with those who remained in the study. The lost to follow-up group only showed a significant decrease in depressed symptoms (*P* = 0.016). Participants provided signed informed permission, and the study's protocol was approved by the National Institute for Development Administration's Human Research Ethics Committee (ECNIDA 2020/00012). Every procedure followed the Declaration of Helsinki and was conducted in compliance with all applicable rules and regulations.

## 3. Measures

### 3.1. Outcome variable

*Bodily pain* in the past month was assessed in 13 body parts (“the head, shoulders, arms, wrists, fingers, chest, abdomen, waist, hips, legs, knees, ankles, and toes”) by using the question, “Did you feel any pain or ache in the following body parts in the last month?” (Response options were “none, mild, moderate, or severe”). We defined NPS with mild, moderate, or severe pain in the past 4 weeks in 13 body parts (0–13),^15,25^ MSP (≥2 pain sites,^16^ and ≥3 pain sites^23^), and the proportion of moderate and severe pain on any of the 13 body parts.

### 3.2. Independent variables

Age, employment, marital status, living situation, and subjective economic status were among the time-varying sociodemographic factors. “How satisfied are you with your economic situation?” was the question used to assess the latter. Higher economic status is indicated by higher scores, which range from 0 to 10. Sex was used in a gender-stratified model, and time-constant factors such as educational attainment, urban/rural residency, and sex were used as sample descriptions.

*Religious involvement* was sourced from the questions, (1) “Making merit and giving alms according to respected religious principles,” (2) “Prayer in the morning/before going to bed,” (3) “Performing merit-making activities at religious places according to the religions that the interviewees respect on important religious days,” and (4) “Observing important religious days that the interviewee respects.” The Cronbach alpha was 0.83, and the response options ranged from 0 = never to 3 = always, for a total range of 0 to 12. Higher scores indicated stronger religious commitment.

*Tobacco smoking* was assessed with the question, “Have you ever smoked cigarettes?” (response options: “1 = yes, and still smoke now; 2 = yes, but quit smoking; and 3 = never”).

*Alcohol use frequency:* 0 = none, 1 = “Drink very little (eg, drink no more than 1 small can of beer or no more than 1 glass of wine/spirits),” 2 = “Drink lightly (eg, drink 1–2 cans of beer or 1–2 glasses of wine/spirits),” 3 = “Drinks quite a bit (eg, drinks approximately 3–4 cans of beer or 3–4 glasses of wine/spirits),” 4 = “Drinks quite a lot (eg, drinks 5–6 cans of beer or 5–6 glasses of wine/spirits) glasses,” and 5 = “Drinks a lot (such as drinking more than 6 small cans of beer or more than 6 glasses of wine/spirits).”

An assessment of one's own physical health was produced using the item, “In general, how would you rate your physical health status?” reported on a “0 (=very poor) to 10 (=excellent)” scale.^26^

The Center for Epidemiologic Studies Depression Scale (CES-D-10) was used to measure depressed symptoms; the Cronbach alpha was 0.69, and the scale's total scores ranged from 0 to 30, with higher scores denoting more depressive symptoms.^4^

In addition to self-reported urinary incontinence from the previous month, healthcare providers diagnosed chronic conditions such as diabetes, hypertension, bone diseases, cardiovascular disease, psychiatric/emotional disorders, brain disease/dementia, liver/gastrointestinal disease, kidney disease, lung disease, prostrate disease, uterine or ovarian diseases, visual impairment, hearing impairment, and cancer.

*Body mass index* was assessed by self-reported body weight and height and classified as obesity (≥30 kg/m^2^).

#### 3.2.1. Data analysis

Descriptive statistics were used to describe the sample. The study waves' percentage differences were computed using χ^2^ and *t*-tests. Linear fixed-effects (FE) regressions and conditional FE logistic regressions were used to estimate the longitudinal associations between time-varying independent variables and time-varying NPS and MSP, respectively, for the 3 study waves from 2015 to 2022. The Hausman specification test between the fixed-effects and random-effects models validated the selection of FE regression (*P* < 0001). Because individual fixed effects account for all person-level variability, this model is particularly effective at reducing biases brought on by systematic relationships between explanatory variables and time-constant factors (both observed and unobserved), such as genetics.^8^ Therefore, the use of FE regressions significantly reduces the issue of unobserved heterogeneity. In accordance with previous reviews, time-varying factors were incorporated.^6,7,17,18^ Sex and education are examples of time-invariant variables that cannot be included as main effects in linear FE regressions. Consequently, education was used for descriptive purposes, and sex, a time-constant component, was used to stratify the linear FE regression models. *P* < 0.05 was deemed significant, and only full cases (missing cases were <4% in study variables, with the exception of body mass index >5%, which was imputed) were examined. We computed cluster-robust standard errors. For statistical analysis, StataSE 16.0 (College Station, TX) was used.

## 4. Results

### 4.1. Sample characteristics

In all, 2450 observations made up the pooled analytical sample: 1038 in wave 1,753 in wave 2, and 661 in wave 3. The average age of the pooled sample was 85.2 years (range: 80–110 years), 44.3% of the participants were men, 62.4% were widowed, 16.4% lacked formal education, 55.1% lived in cities, and 15.3% were living alone. The average NPS was 2.4 (SD = 3.1); the proportion of MSP was 46.1% and 31.4% for having ≥2 and ≥3 pain sites, respectively; and 42.8% reported moderate or severe pain on at least 1 pain site. Educational level differed by sex, and residence status did not differ by sex. Independent variables, including age, work, and marital status, smoking and alcohol use, self-rated physical health status, number of chronic conditions, and obesity differed by sex. However, there was no sex-based difference in subjective economic status, living alone, religious activity, or depressed symptoms (Table 1).

**Table 1:** Analytic sample characteristics; pooled waves (1, 2, and 3); health, aging, and retirement in Thailand 2015 to 2022.

Variable (range)	Total	Male	Female	*P* *
	N = 2450	N = 1085	N = 1365	
	*N* (%)/Mean (SD)	*N* (%)/Mean (SD)	*N* (%)/Mean (SD)	
No. of pain sites (0–13): M (SD)	2.4 (3.1)	2.3 (3.0)	2.4 (3.1)	0.791
≥2 pain sites: *N* (%)	1139 (46.1)	496 (45.6)	643 (46.5)	0.654
≥3 pain sites: *N* (%)	776 (31.4)	342 (31.6)	434 (31.4)	0.978
Age (80–110 y): M (SD)	85.2 (4.5)	85.0 (4.2)	85.4 (4.7)	0.015
Marital status: *N* (%)				
Single/divorced/separated/married	912 (37.6)	655 (60.7)	257 (19.5)	<0.001
Widowed	1512 (62.4)	424 (39.3)	1088 (80.9)	
Education: *N* (%)				
None	400 (16.4)	109 (10.1)	291 (21.4)	<0.001
Primary	1894 (77.6)	867 (80.4)	1027 (75.4)	
>Primary	146 (6.0)	103 (9.5)	43 (3.2)	
Subjective economic status (0–10): M (SD)	6.5 (2.0)	6.6 (1.9)	6.5 (2.0)	0.077
Work status (working): *N* (%)	174 (7.5)	103 (10.1)	71 (5.4)	<0.001
Residence (urban): *N* (%)	1350 (55.1)	604 (55.7)	746 (54.7)	0.595
Living alone: *N* (%)	372 (15.3)	169 (15.7)	203 (14.9)	0.717
Religious involvement (0–12): M (SD)	6.3 (3.7)	6.2 (3.8)	6.4 (3.6)	0.231
Smoking: *N* (%)				
Never	2003 (82.0)	693 (64.2)	1310 (96.1)	<0.001
Past	311 (12.7)	268 (24.8)	43 (3.2)	
Now	129 (5.3)	119 (11.0)	10 (0.7)	
Alcohol use frequency (0–5): M (SD)	0.14 (1.8)	0.27 (2.7)	0.03 (0.6)	<0.001
Self-rated physical health (0–10): M (SD)	6.57 (2.0)	6.66 (1.9)	6.50 (2.0)	0.035
Depressive symptoms (0–30): M (SD)	5.5 (3.8)	5.46 (3.8)	5.55 (3.8)	0.566
No. of chronic conditions (0–15): M (SD)	1.78 (1.5)	1.71 (1.5)	1.84 (1.4)	0.028
Obesity: *N* (%)	82 (3.4)	27 (2.5)	55 (4.0)	0.028

* Based on χ^2^ tests or *t*-tests or ANOVA.

### 4.2. Determinants of the number of pain sites

The results of the linear FE regression showed a significant correlation between increasing age and decreasing NPS in the overall sample (β −0.25, *P* < 0.001), and in men (β −0.21, *P* < 0.001) and women (β −0.28, *P* < 0.001). An increase in subjective economic status was associated with decreasing NPS in the total sample (β −0.13, *P* = 0.031) and among women (β −0.16, *P* = 0.042). Work status decreased the NPS in the total sample (β −0.97, *P* = 0.037), and among men (β −1.38, *P* = 0.029) and women (β −1.51, *P* = 0.033). Transitioning to living alone was associated with an increasing NPS in the overall sample (β 0.74, *P* = 0.035) and in women (β 1.03, *P* = 0.038). Furthermore, increases in depressive symptoms were significantly associated with a higher NPS in the total sample (β 0.19, *P* < 0.001), among men (β 0.14, *P* < 0.001), and among women (β 0.15, *P* < 0.001). Past smoking was positively associated with the NPS in the total sample (β 0.85, *P* = 0.023) and among women (β 2.06, *P* = 0.021). In addition, among women, increases in the number of chronic conditions were associated with increases in the NPS (β 0.19, *P* = 0.039), and among men, increases in religious involvement decreased the NPS (β −0.14, *P* = 0.013) (Table 2).

**Table 2:** Determinants of the number of pain sites from linear fixed-effects regression analyses (waves 1, 2, and 3).

Independent variables	All		Male		Female	
	b (SE)	*P*	b (SE)	*P*	b (SE)	*P*
Age	−0.25 (0.03)	<0.001	−0.21 (0.05)	<0.001	−0.28 (0.04)	<0.001
Marital status						
Single/divorced/separated/married	Reference		Reference		Reference	
Widowed	−0.57 (0.42)	0.173	−0.68 (0.60)	0.255	−0.47 (0.61)	0.439
Subjective economic status	−0.13 (0.06)	0.031	−0.13 (0.10)	0.170	−0.16 (0.08)	0.042
Work status (working)	−0.97 (0.46)	0.037	−1.38 (0.63)	0.029	−1.51 (0.71)	0.033
Living arrangement						
Living with others	Reference		Reference		Reference	
Living alone	0.74 (0.24)	0.035	0.81 (0.52)	0.117	1.03 (0.50)	0.038
Religious involvement	−0.08 (0.04)	0.052	−0.14 (0.07)	0.013	−0.07 (0.06)	0.243
Smoking						
Never	Reference		Reference		Reference	
Past	0.85 (0.37)	0.023	0.51 (0.42)	0.228	2.06 (0.89)	0.021
Now	0.31 (0.67)	0.647	−0.04 (0.75)	0.962	0.78 (1.54)	0.612
Alcohol use frequency	−0.04 (0.19)	0.818	0.11 (0.38)	0.779	−0.12 (0.22)	0.583
Self-rated physical health	−0.07 (0.06)	0.254	−0.07 (0.07)	0.326	−0.12 (0.08)	0.142
Depressive symptoms	0.19 (0.03)	<0.001	0.14 (0.03)	<0.001	0.15 (0.04)	<0.001
No. of chronic conditions	0.09 (0.07)	0.168	0.09 (0.18)	0.616	0.19 (0.09)	0.039
Obesity	−0.59 (0.73)	0.416	1.25 (1.16)	0.280	−1.63 (0.95)	0.086
Constant	11.50 (1.79)	<0.001	10.29 (2.99)	<0.001	12.49 (2.31)	<0.001
Observations	2450		1085		1365	
Individuals	1676		749		938	
*R* ^2^	0.17		0.16		0.22	

Unstandardized regression coefficients of fixed-effects regression analysis with robust standard errors in parentheses.

### 4.3. Determinants of multisite pain

Fixed-effects logistic regressions showed that the probability of having ≥2 and ≥3 pain sites decreased with age (odds ratio [OR]: 0.81, 95% confidence interval [CI]: 0.77–0.86, and OR: 0.77, 95% CI: 0.72–0.83, respectively). Higher subjective economic status decreased having ≥2 and ≥3 pain sites (OR: 0.85, 95% CI: 0.78–0.93, and OR: 0.88, 95% CI: 0.80–0.97, respectively). Transitioning to live alone increased having ≥3 pain sites (OR: 1.94, 95% CI: 1.17–3.24). Past smoking increased the odds of having ≥2 and ≥3 pain sites (OR: 1.97, 95% CI: 1.13–3.42, and OR: 1.83, 95% CI: 1.03–3.25, respectively). Depressive symptoms increased the odds of having ≥2 and ≥3 pain sites (OR: 1.11, 95% CI: 1.06–1.16, and OR: 1.13, 95% CI: 1.07–1.19, respectively), and the number of chronic conditions increased the odds of having ≥2 and ≥3 pain sites (OR: 1.21, 95% CI: 1.00–1.45, and OR: 1.22, 95% CI: 1.01–1.40, respectively) (Table 3).

**Table 3:** Determinants of multisite pain from conditional fixed-effects logistic regression analyses (waves 1, 2, and 3).

Independent variables	≥2 pain sites		≥3 pain sites	
	OR (95% CI)	*P*	OR (95% CI)	*P*
Age	0.81 (0.77–0.86)	<0.001	0.77 (0.72–0.83)	<0.001
Marital status				
Single/divorced/separated/married	Reference		Reference	
Widowed	0.77 (0.41–1.45)	0.422	0.70 (0.37–1.32)	0.271
Subjective economic status	0.85 (0.78–0.93)	<0.001	0.88 (0.80–0.97)	0.006
Work status (working)	0.99 (0.49–2.02)	0.982	0.68 (0.33–1.37)	0.275
Living arrangement				
Living with others	Reference		Reference	
Living alone	1.39 (0.83–2.33)	0.210	1.94 (1.17–3.24)	0.011
Religious involvement	0.99 (0.93–1.06)	0.819	0.98 (0.91–1.05)	0.530
Smoking				
Never	Reference		Reference	
Past	1.97 (1.13–3.42)	0.017	1.83 (1.03–3.25)	0.039
Now	1.23 (0.49–3.08)	0.659	1.98 (0.64–6.13)	0.237
Alcohol use frequency	1.58 (0.87–2.85)	0.133	1.70 (0.81–3.59)	0.161
Self-rated physical health	0.97 (0.89–1.06)	0.548	0.93 (0.85–1.02)	0.111
Depressive symptoms	1.11 (1.06–1.16)	<0.001	1.13 (1.07–1.19)	<0.001
No. of chronic conditions	1.21 (1.00–1.45)	0.046	1.22 (1.01–1.40)	0.041
Obesity	0.88 (0.31–2.47)	0.808	0.43 (0.14–1.34)	0.147
Observations	803		733	
Individuals	336		305	
*R* ^2^	0.19		0.20	

CI, confidence interval; OR, odds ratio.

## 5. Discussion

This study's objective was to use national, longitudinal community-dwelling data from 2015 to 2022 to determine for the first time the factors that influence the NPS and MSP among Thailand's very elderly (those 80 years and older). Regarding the NPS, FE regressions showed that increasing age, subjective economic status, and work status decreased the NPS. Transitioning to live alone, past smoking, and increase in depressive symptoms increased the NPS. In addition, among women, an increase in the number of chronic conditions increased the NPS, and among men, an increase in religious involvement was associated with a decrease in the NPS. Regarding FE regressions with MSP (≥2 and ≥3 pain sites), age and subjective economic status decreased the odds of MSP, transitioning to live alone, past smoking, depressive symptoms, and the number of chronic conditions increased the odds of MSP.

The reported moderate or severe pain on at least 1 pain site (42.8%) and MSP (≥2 pain sites: 46.1%) in this study compares with a study in Sweden (for ≥2 MSP 46% and for severe pain in at least 1 location 35%),^7^ but was lower than in North Rhine-Westphalia, Germany (45.1% moderate pain and 26.4% severe pain),^6^ and higher than in a multisite study in Germany (33% mild to severe pain).^17^ Some of these differences may be related to different pain measures and sociocultural differences in the study countries.^6^

Consistent with studies in Sweden^7^ and Germany,^6,17^ increasing age was associated with decreases in the NPS and MSP. Possible reasons for this could be related to how ageing affects the experience of pain, a reduced ability to detect harmful signals implicated in the processing of painful stimuli, and the threshold at which pain is experienced could increase with age.^20^ For example, the heat pain threshold in older persons increases compared with that of younger adults, demonstrating a decrease of heat pain sensitivity.^35^ We found that transitioning to living alone increased NPS and MSP. Living alone may be associated with an increase in depressive symptoms, loneliness, and insomnia,^5,27^ all of which may contribute to an increase in NPS and MSP. Previous research with older adults showed lower income^1,15^ and being unemployed among men^1^ was associated with NPS and/or MSP. These findings (lower subjective economic status and not working) are consistent with our results being associated with NPS and MSP among the oldest-old.

Regarding health-related factors, we found in line with previous research^17^ that an increase in depressive symptoms was linked with increases in NPS and MSP, as well as a higher number of chronic diseases^6,18^ increased NPS and MSP. Serotonin and norepinephrine are the neurotransmitters that affect both depression and pain, suggesting that there is more to physical pain and depression than just cause and effect. Depression and pain are associated with these transmitters' dysregulation.^32^ There is likely to be a bidirectional relationship between depression and pain.^19^ Multimorbidity, having several conditions at the same time, may increase the likelihood of having pain in a higher NPS.^6,10^ As the extent of multimorbidity was higher in women than in men in this study, this may explain the preponderance of the association between an increase in chronic conditions and an increase in NPS in oldest-old women.

We did not, however, find an association between increases in obesity and increases in NPS, as was found in a previous systematic review among adults.^29^ It is possible that the low prevalence of obesity (<3.5%) and the self-reported assessment of body weight and height contributed to this nonsignificant finding. Poorer self-rated health status was found to be associated with increases in pain,^6^ while this result was not found in this study. Previous longitudinal research among adults showed a link between former and current smoking status and increased risk of CWP and NPS,^21,22^ and alcohol intake lowered the risk of CWP,^21,22^ while this study showed a significant association between ex-smokers and increase in NPS and MSP, and alcohol use frequency was not significantly related to increases in NPS and MSP. The association between ex-smokers and NPS and MSP may be complex because of possible mediating factors, such as depression.^12,21^ Previous research with older adults showed that lack of physical activity^15,21^ was associated with NPS, while in our study, religious involvement (which may include physical activity) was protective against NPS.

### 5.1. Study strengths and limitations

A nationwide, longitudinal community-dwelling population is included in the study, and HART used validated metrics from the Korean Longitudinal Study of Aging and the Health and Retirement Study. Using panel FE regression models, we were able to reduce the problem of unobserved heterogeneity. The fact that all data, including body weight and height, were evaluated using self-report is 1 study drawback; objective measurements should be included in future evaluations. A fairly good response rate was achieved. This study's attrition analysis revealed no discernible variations in most health, social, and sociodemographic factors. We only were able to assess body pain in 2015, 2017, and 2022, because in the 2020 cohort, body pain was not assessed. Some of the variables that could influence NPS and MSP, such as frailty,^24^ use of analgesics,^17^ and cognitive function,^17,18^ were not assessed and should be included in future studies. Finally, the sample did not include oldest-old adults living in institutional or assisted living communities, which may have yielded higher NPS and MSP rates than in community-dwelling oldest-old adults.

## 6. Conclusions

Decreasing age, decreasing subjective economic status, not working, living alone, past smoking, depressive symptoms, and chronic conditions were associated with the NPS and/or MSP. Strategies to reduce living alone, improve economic status, delay or decrease depressive symptoms, and manage chronic conditions may help to reduce NPS and MSP in this unique population group.

## Disclosures

The authors have no conflict of interest to declare.
